# High cholesterol triggers white matter alterations and cognitive deficits in a mouse model of cerebrovascular disease: benefits of simvastatin

**DOI:** 10.1038/s41419-018-1199-0

**Published:** 2019-01-28

**Authors:** Xin-Kang Tong, Lianne J. Trigiani, Edith Hamel

**Affiliations:** Laboratory of Cerebrovascular Research, Montreal Neurological Institute, McGill University, 3801 University Street, Montréal, H3A 2B4 QC Canada

## Abstract

Transgenic mice overexpressing transforming growth factor-β1 (TGF mice) display impaired cerebrovascular reactivity, cerebral hypoperfusion and neurovascular uncoupling, but no overt cognitive deficits until old age. Cardiovascular diseases are a major risk factor for vascular cognitive impairment and dementia (VCID). We investigated the impact of a high cholesterol diet (HCD) on cerebrovascular and cognitive function in adult (6 months) and aged (12 months) TGF mice, together with the potential benefit of simvastatin (SV), an anti-cholesterol drug with pleiotropic effects, in adult mice. HCD increased blood, but not brain, cholesterol levels in treated mice, which SV did not reduce. In WT mice, HCD induced small, albeit significant, impairment in endothelium-dependent dilatory function. In TGF mice, HCD worsened the established brain vessel dilatory dysfunction in an age-dependent manner and increased the number of string vessels in the white matter (WM), alterations respectively normalized and significantly countered by SV. HCD triggered cognitive decline only in TGF mice at both ages, a deficit prevented by SV. Concurrently, HCD upregulated galectin−3 immunoreactivity in WM microglial cells, a response significantly reduced in SV-treated TGF mice. Grey matter astrogliosis and microgliosis were not affected by HCD or SV. In the subventricular zone of adult HCD-treated TGF mice, SV promoted oligogenesis and migration of oligodendrocyte progenitor cells. The results demonstrate that an underlying cerebrovascular pathology increases vulnerability to cognitive failure when combined to another risk factor for dementia, and that WM alterations are associated with this loss of function. The results further indicate that myelin repair mechanisms, as triggered by SV, may bear promise in preventing or delaying cognitive decline related to VCID.

## Introduction

Alzheimer’s disease (AD) and vascular cognitive impairment and dementia (VCID), the two most common forms of dementia in the aging population, are both heterogeneous and multifaceted^1^. VCID is characterized as a progressive cognitive decline attributable to cerebrovascular factors^1–3^. VCID has been associated with increased cerebral blood vessel thickness and stiffness (vascular fibrosis)^4,5^, endothelial dysfunction, and small vessel disease. These impairments result in chronically reduced cerebral perfusion leading to shortage of oxygen and nutrients supply to the brain parenchyma, with a high vulnerability of the white matter (WM) particularly in VCID related to small vessel disease^1–3^. The traditional risk factors for heart disease and stroke, such as diabetes, hypercholesterolemia, hypertension, obesity, and sedentariness are also the main risk factors for both VCID and AD^1–3^.

A commonality of VCID and AD is the presence of an inflammatory response, which likely plays a key role in the development and progression of WM lesions and neuronal loss, leading to learning and memory deficits. In this respect, altered levels of the multifunctional cytokine transforming growth factor-β1 (TGF-β1) are found in brain, plasma, cerebrospinal fluid or brain vessels of both AD and VCID patients^6–8^. Additionally, impaired TGF-β1 signaling was reported in various forms of small vessel diseases^9,10^, and TGF-β1 polymorphisms have been associated with VCID^11^ or with an increased risk for VCID and AD^12,13^.

Interestingly, transgenic mice that overexpress a constitutively active form of TGF-β1 (TGF mice) in brain display a cerebrovascular pathology that includes vascular fibrosis characterized by accumulation of structural proteins in, and thickening of, the vessel basement membrane, smaller capillary endothelial cells and pericytes, degenerating capillaries^14^ and, ultimately, a string vessel pathology^15,16^ characterized by loss on capillary endothelial cells, capillary remnants or intercapillary bridges^17^. These changes are accompanied by impaired cerebrovascular reactivity, chronic cerebral hypoperfusion^18^, and impaired neurovascular coupling^15,16^. Such alterations recapitulate particularly well those seen in VCID^15^ and, except for the cerebral amyloid angiopathy^19^, in AD^14^. Yet, despite impaired cerebrovascular function and increased astroglial TGF-β1 production and secretion that can affect brain homeostasis through signaling alterations in different cellular compartments, TGF mice display no or subtle^15,16,20,21^ cognitive deficits even late in age. This raised the possibility that a compromised cerebral circulation may promote cognitive failure when combined with another risk factor for dementia^15^. Therein, we tested this hypothesis in adult and aged TGF mice rendered or not hypercholesterolemic and, in adult mice, we further tested the potential benefits of the anti-cholesterol drug simvastatin (SV) known for its pleiotropic effects on the brain vasculature^15,22^, neuronal function^22,23^, and WM^24^.

## Results

### High cholesterol diet (HCD) increased blood, but not brain, cholesterol levels: effects of simvastatin (SV)

HCD increased total blood cholesterol levels more than two-fold in adult and aged WT and TGF mice compared to mice fed a normal diet (Supplementary Table 1). In blood, low-density lipoprotein (LDL) cholesterol was low in control WT and TGF mice, but dramatically increased in HCD-treated groups. High-density lipoprotein (HDL) cholesterol slightly increased in HCD-treated mice, and there was no change in the levels of blood triglycerides between any of the groups (Supplementary Table 1). SV did not affect blood total cholesterol levels in WT or TGF mice fed a HCD, and it had negligible or no effects on blood LDL, HDL, and triglycerides (Supplementary Table 1). In brains of adult TGF mice, neither HCD nor SV affected total cholesterol levels (Supplementary Table 2).

### HCD induced cognitive deficits selectively in TGF mice: effects of SV

**Fig. 1:**
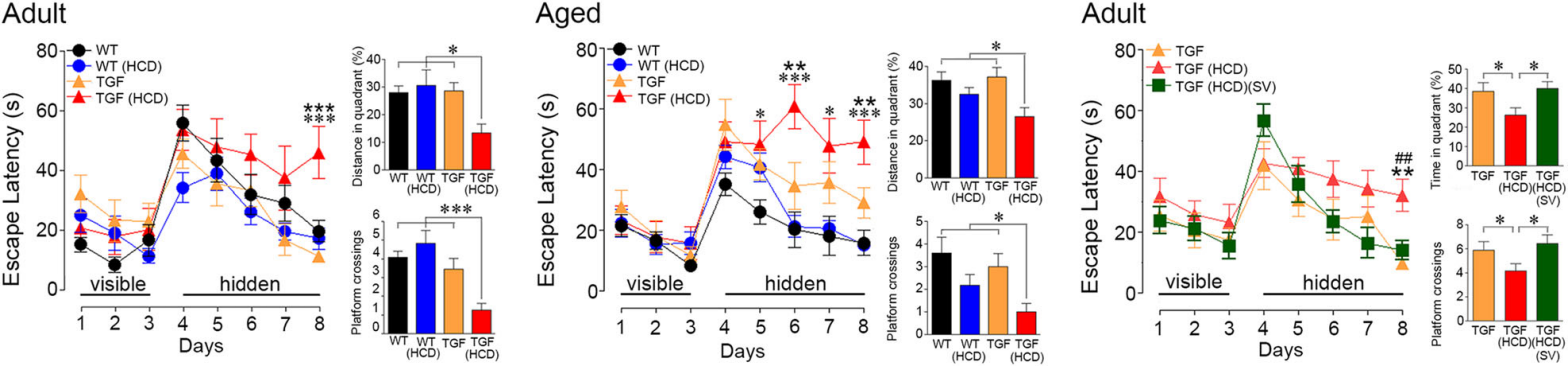
Effects of high cholesterol diet (HCD) on spatial learning. Adult and aged TGF mice treated with HCD (red triangle) displayed impaired learning during hidden-platform testing in the Morris water maze compared to all other groups of mice. These deficits were not due to visual or motor disabilities as HCD-fed TGF mice performed as well as the other groups in finding the visible platform (days 1–3). During the probe trail, both adult and aged HCD-treated TGF mice spent shorter time in the target quadrant and had less crossings over the previous location of the platform. Simvastatin (SV) treatment improved learning and memory in the adult TGF fed a HCD (green square). Error bars represent SEM (*n* = 9–13 mice/group). *Compared to WT; : compared to TGF; # compared to TGF(HCD)(SV). *p < 0.05; , ##: *p* < 0.01; ***, : *p* < 0.001 using two-way ANOVA and repeated measures ANOVA followed by Newman-Keuls post-hoc test

WT and TGF mice were as effective in finding the visible platform (Fig. 1), indicating no visual, motor or motivation deficits. In the hidden platform testing, adult TGF mice performed as well as WT controls, whereas aged TGF mice exhibited slightly, albeit not significant, longer latency time to find the platform (Fig. 1). HCD induced learning deficits in both adult and aged TGF mice. Adult HCD-fed TGF mice differed from the other groups only on the last day of hidden platform testing, whereas aged HCD-fed TGF mice needed more time to find the platform compared to all other groups on most days of testing (Fig. 1). During the probe trial, adult and aged TGF mice performed as well as age-matched control WT mice (Fig. 1). In contrast, HCD-fed TGF mice, irrespective of age, showed deficits compared to all groups, as shown by a shorter time spent in the target quadrant and less crossings over the previous location of the platform (Fig. 1). Learning and memory performances of adult and aged WT control mice were not affected by HCD. In adult TGF mice, cognitive deficits induced by HCD were prevented by SV, SV HCD-treated mice performing as well as control TGF mice (Fig. 1, right panel).

### Age-dependent impairments of HCD on cerebrovascular function in TGF mice: effects of SV

**Fig. 2:**
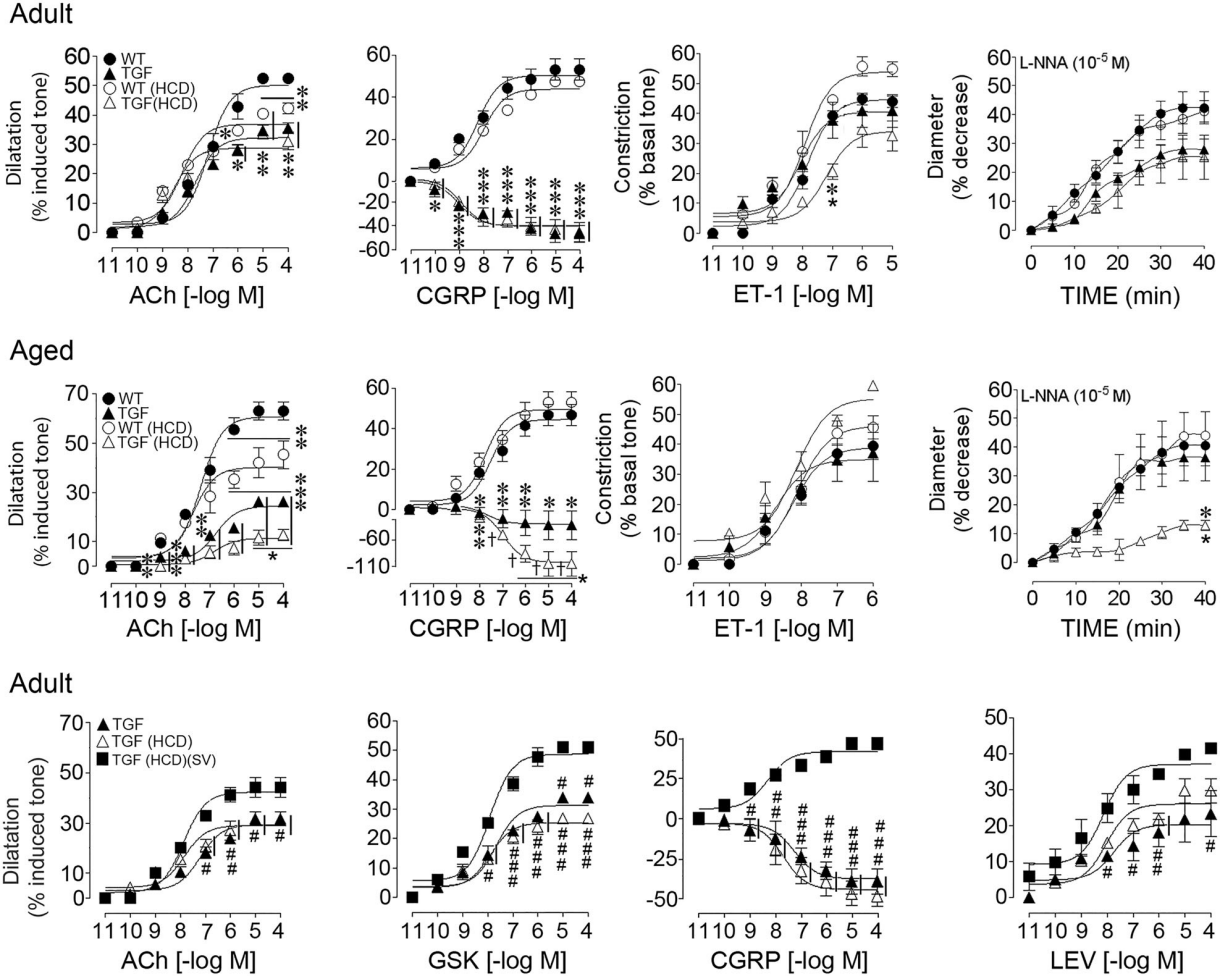
Effects of high cholesterol diet (HCD) on cerebrovascular reactivity in adult and aged mice: recovery by simvastatin (SV). Top panel: Cerebrovascular responses of the posterior cerebral artery (PCA) to ACh, CGRP, ET-1 and the NOS inhibitor L-NNA were measured in adult WT (solid circle) and TGF (solid triangle) mice. In TGF mice, ACh-induced and CGRP-induced dilations were significant decreased, the dilatory response to CGRP being even reversed to constriction. HCD had minor effects in adult TGF mice (open triangle) resulting in a modest, albeit significant, reduction in 10^–7^ M ET-1-induced contraction, without worsening effects on the dilatory function or slightly reduced baseline NO synthesis. HCD reduced the ACh-mediated dilation in adult WT mice. Middle panel: Aged TGF mice treated with a HCD (open triangle) showed significant worsening of the already impaired ACh-induced and CGRP-induced dilations seen in TGF mice fed a standard diet (solid triangle), and displayed highly reduced NO production. HCD selectively impaired ACh-mediated dilation in WT mice (open circle), and more severely so than in adult WT mice. Bottom panel: In adult TGF (solid triangle) and HCD-treated TGF (open triangle) mice, ACh-induced and CGRP-induced dilations were similarly impaired, and concurrent treatment with simvastatin (SV) in HCD-fed TGF mice (solid square) normalized both dilatory responses. Further, SV restored TRPV4 channel-mediated dilation and improved KATP channel function, measured with their respective channel opener GSK1016790A (GSK) and levcromakalim (LEV). Vertical bars besides groups and horizontal bars under concentrations indicate similar significance. * or †: compared to WT; : compared to TGF; #: compared to TGF(HCD)(SV). Error bars represent SEM. *, , #*p* < 0.05; **, ##: *p* < 0.01; ***, ###, †: *p* < 0.001 using two-way ANOVA and repeated measures ANOVA followed by Newman-Keuls post-hoc test

**Fig. 3:**
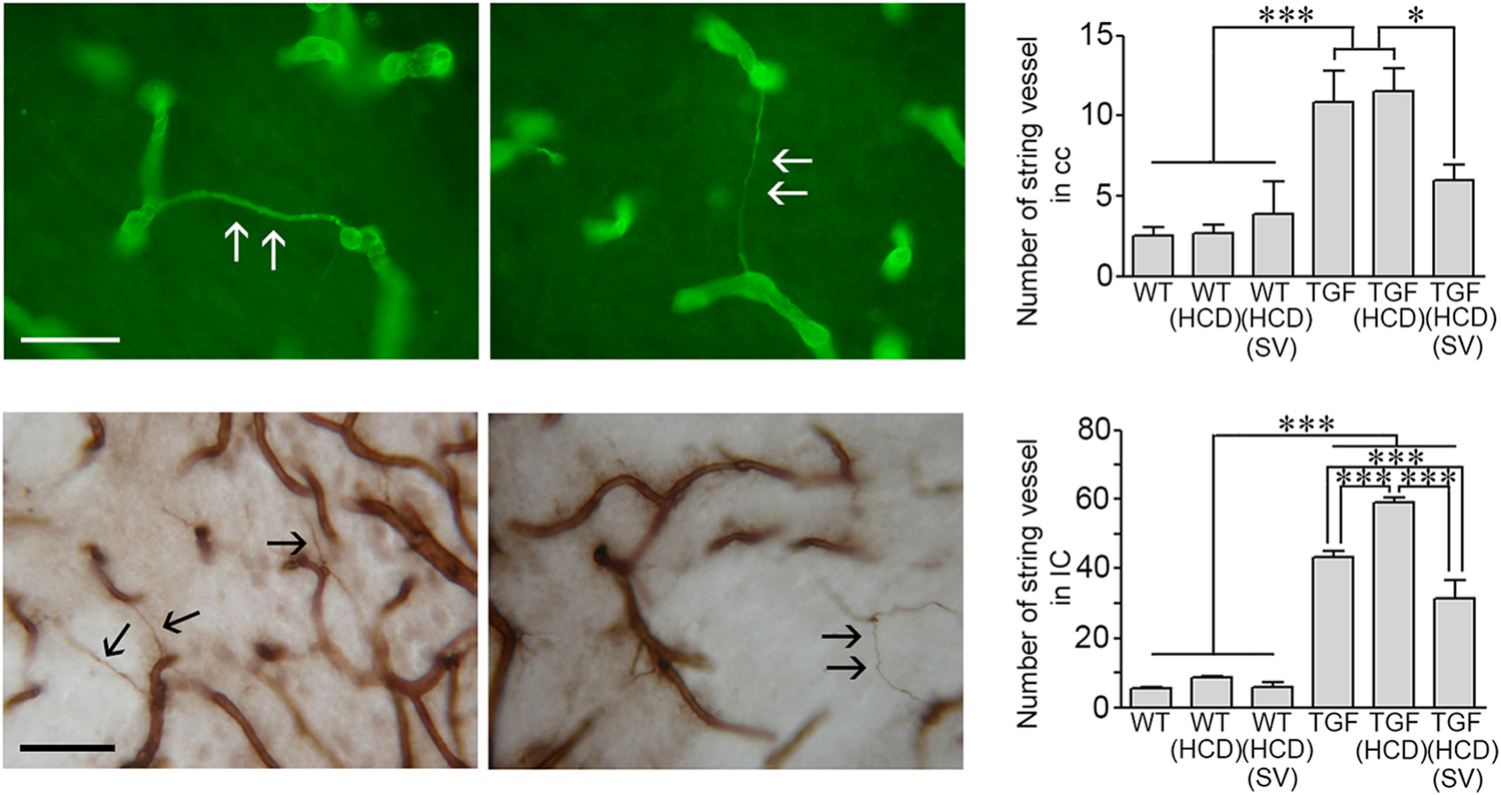
String vessel pathology in the white matter: Effects of high cholesterol diet (HCD) and simvastatin (SV) treatment. String vessels visualized with collagen IV immunofluorescence (Cy2, green) (top panel) or DAB immunohistochemistry (brown, bottom panel) appear as small ribbons (arrows) between normal capillaries. In adult TGF mice, the number of string vessels in the corpus callosum (cc) and internal capsule (IC) was significantly increased compared to WT mice. HCD further increased this number only in the IC. SV effectively reduce this pathology in both cc and IC in HCD-fed TGF mice. *p < 0.05, ***p < 0.001 using two-way ANOVA followed by Newman-Keuls post-hoc test. Bar: 30 μm

TGF mice of both ages displayed impaired dilations to acetylcholine (ACh) and calcitonin gene-related peptide (CGRP) compared to WT controls, CGRP dilations being even reversed to contractile responses. However, there were no significant alterations in their contractile response to endothelin−1 (ET-1) or basal nitric oxide (NO) bioavailability following nitric oxide synthase (NOS) inhibition with N^ω^-nitro-L-arginine (L-NNA) (Fig. 2, Table 1). HCD had minor effects in adult TGF mice resulting in modest impairments in ET-1-mediated contractions, without worsening the already present dilatory deficits (Fig. 2, Table 1). In contrast, in aged TGF mice, HCD worsened the existing impaired dilatory responses to both ACh and CGRP, and it significantly reduced the contractile response to NOS inhibition, without altering ET-1 contractile responses (Fig. 2, Table 1). In adult and aged WT controls, HCD induced selective impairments in endothelial-mediated ACh dilations (Fig. 3), aged WT mice being more severely affected (Fig. 2, Table 1). None of the deficits were attributed to receptor desensitization since ACh and CGRP pD_2_ values did not differ between groups (Table 1).

**Table 1:** (a) Effect of high cholesterol diet (HCD) on cerebrovascular reactivity in adult mice

		WT (3)	TGF (4)	WT (HCD) (3)	TGF (HCD) (3)
ACh	(Emax)	50.2 ± 2.03	32.3 ± 1.10***	36.9 ± 1.48***	28.8 ± 1.32***
	(pD2)	7.19 ± 0.13	7.68 ± 0.13*	8 .22 ± 0.16**	8.54 ± 0.20**
CGRP	(Emax)	50.3 ± 2.25	−2.9 ± 4.60***	48.8 ± 1.56	−1.2 ± 4.54***
	(pD2)	8.20 ± 0.19	8.99 ± 0.30	7.96 ± 0.14	8.9 ± 0.28
ET-1	(Emax)	44.7 ± 1.6	40.7 ± 2.71	53.9 ± 2.42*	34.1 ± 2.74*
	(pD2)	7.82 ± 0.11	8.06 ± 0.24	7.93 ± 0.15	7.21 ± 0.24
L-NNA	(Emax)	37.3 ± 2.09	25.9 ± 1.44**††	34.9 ± 1.76	23.3 ± 2.26**††
(b) Effect of high cholesterol diet (HCD) on cerebrovascular reactivity in aged mice					

		WT (3)	TGF (3)	WT (HCD) (3)	TGF (HCD) (3)
ACh	(Emax)	60.7 ± 2.24	24.4 ± 1.30***†††	40.1 ± 2.51***	11.4 ± 1.22***†††
	(pD2)	7.37 ± 0.13	6.75 ± 0.17	7.67 ± 0.24	6.81 ± 0.33
CGRP	(Emax)	44.5 ± 2.34	1.0 ± 9.37***††	49.4 ± 2.67	−5.2 ± 6.47***†††
	(pD2)	7.51 ± 0.19	7.70 ± 0.87	7.73 ± 0.21	6.98 ± 0.23
ET-1	(Emax)	38.9 ± 2.46	35.0 ± 3.69	46.2 ± 2.32	55.3 ± 3.59*
	(pD2)	8.24 ± 0.18	8.72 ± 0.36	8.11 ± 0.14	8.15 ± 0.20
L-NNA	(Emax)	32.4 ± 2.68	37.34 ± 3.19	33.6 ± 2.33	12.7 ± 1.43***†††
(c) Effect of simvastatin on cerebrovascular reactivity in adult TGF mice treated with HCD					

		TGF (4)	TGF (HCD) (3)	TGF(HCD)(SV) (4)	
ACh	(Emax)	29.4 ± 1.23	29.5 ± 1.54	42.3 ± 1.48∞∞∞	
	(pD2)	7.21 ± 0.15	7.85 ± 0.22	7.87 ± 0.13	
CGRP	(Emax)	−2.7 ± 3.35	−2.9 ± 2.79	34.9 ± 1.52∞∞∞	
	(pD2)	7.69 ± 0.31	7.26 ± 0.24	8.30 ± 0.19	
GSK	(Emax)	29.8 ± 1.64	24.8 ± 1.34	47.6 ± 1.93∞∞∞	
	(pD2)	7.79 ± 0.19	7.97 ± 0.14	7.96 ± 0.19	
LEV	(Emax)	20.4 ± 2.16	26.1 ± 1.50	37.1 ± 1.88∞∞	
	(pD2)	7.75 ± 0.49	8.04 ± 0.24	8.13 ± 0.24	

Data are means ± SEM of the number of mice indicated within parentheses, and are expressed as the maximal agonist response (EAmax) or potency (pD2: −[log EC50 value]. EAmax is the percentage of maximal dilation to acetylcholine (ACh), calcitonin gene-related peptide (CGRP), that TRPV4 channel opener GSK1016790A (GSK) and the KATP channel opener levcromakalim (LEV) or the maximal percentage decrease in the diameter of arteries to ET or incubated (40 min) with the NOS inhibitor L-NNA (10–5 M). *p < 0.05, **p < 0.01, ***p < 0.001 when compared to WT; *p* < 0.01; *p* < 0.001 when compared to TGF; ††*p* < 0.01; †††*p* < 0.001 when compared to WT (HCD); ∞∞*p* < 0.01; ∞∞∞*p* < 0.001 when compared to TGF (HCD) by two-way ANOVA (A, B) or one-way ANOVA (C) followed by a Newman-Keuls post-hoc analysis

In adult HCD-fed TGF mice, SV improved ACh-mediated and CGRP-mediated dilations (Fig. 2, bottom panel), maximal responses being comparable to those found in similarly aged WT control mice (Fig. 2, top panel, and Table 1). When using the channel opener GSK1016790A to investigate endothelial transient receptor potential vanilloid type 4 (TRPV4) channels that mediate ACh-induced dilations in mouse brain arteries and that are impaired in TGF mice^25^, we found no aggravating effect in HCD-fed TGF compared to untreated TGF mice. SV fully restored this deficit in adult HCD-fed TGF mice (Fig. 2, bottom panel) with maximal responses being comparable to those for ACh in similarly aged WT mice (Table 1). ATP-sensitive K^+^ (KATP) channels mediate a large proportion of CGRP-induced dilations in mouse brain arteries^15^, and dilations induced by the selective KATP channel opener levcromakalim were similarly reduced in vessels from adult TGF mice fed or not a HCD (Fig. 2, Table 1). SV significantly improved KATP channel function in vessels from HCD-fed mice (Fig. 2, *p* < 0.05, Table 1). In a subset of adult TGF mice in which whisker-evoked neurovascular coupling responses were measured, untreated TGF mice displayed the expected impaired CBF increase (7.91 ± 1.13 in TGF vs 14.41 ± 2.21% in WT mice)^15,16^. The latter was not worsened by a HCD (↓24%, n.s.), but it was significantly improved (↑44%, *p* < 0.05) in adult HCD-fed TGF mice treated with SV (6.02 ± 1.03 in HCD-fed TGF mice vs 10.74 ± 1.01 in those treated with SV).

### String vessel pathology in TGF mice: effects of HCD and SV

String vessels correspond to degenerating capillaries with no endothelial cell layer but only a thin basal membrane that can be immunodetected with collagen IV^17^. HCD significantly increased the number of string vessels in the hippocampus (29.0 ± 4.2 vs 44.3 ± 4.3, *p* < 0.01), but not cerebral cortex (11.3 ± 2.0 vs 12.7 ± 3.5, ns) of adult TGF mice, having no effect in WT mice that displayed low number of these vessels (*n* < 10) in both regions. When focusing on the WM, only a few string vessels were found in the corpus callosum (cc) and internal capsule (IC) of WT mice (Fig. 3). This number significantly increased in TGF mice, a pathology exacerbated in the IC of HCD-fed TGF mice (Fig. 3). In adult HCD-fed TGF mice, SV significantly reduced the number of string vessels in both WM areas (Fig. 3), but not in hippocampus (60.0 ± 3.4 in HCD-TGF vs 53.5 ± 3.1 in SV treated HCD-TGF mice).

### HCD selectively affects microglial cells in the WM: effects of SV

**Fig. 4:**
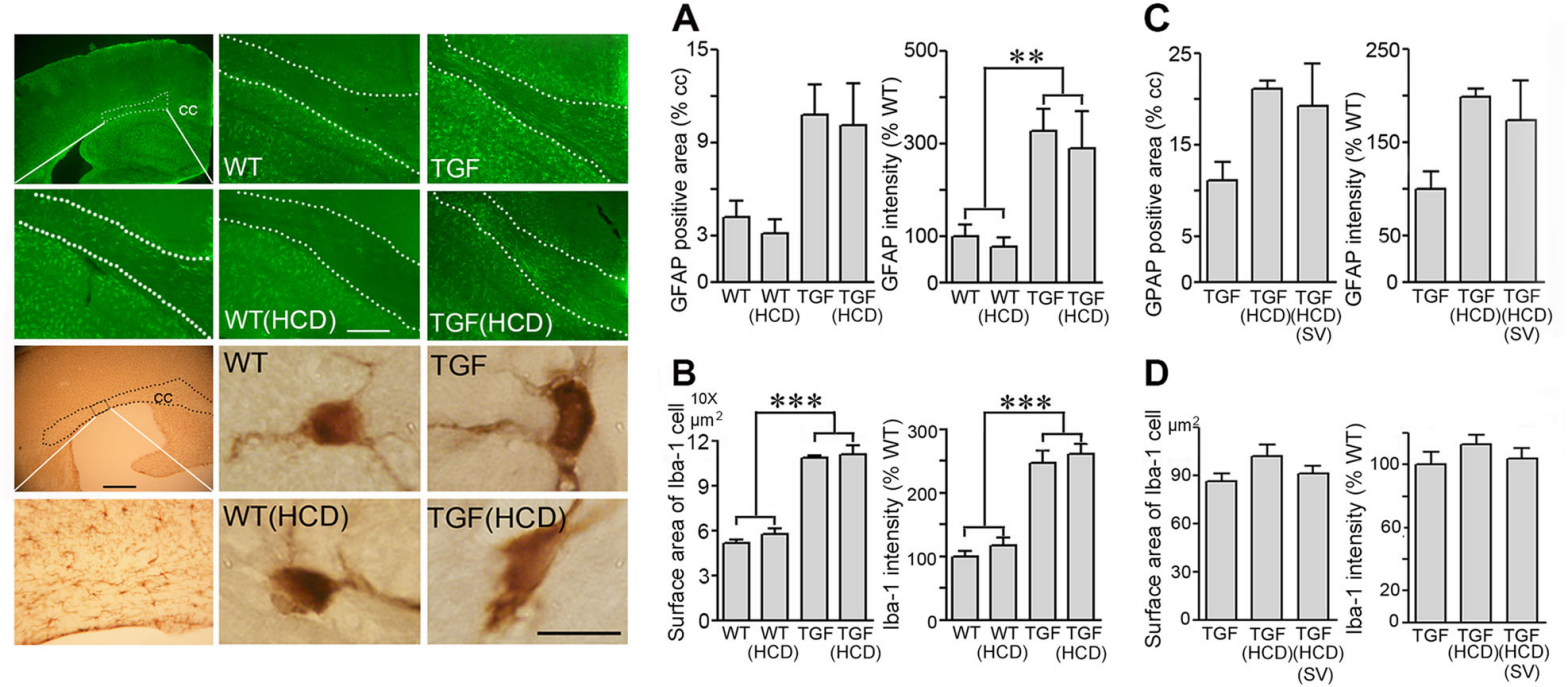
Effects of high cholesterol diet (HCD) and simvastatin (SV) on white matter astrgliosis and microgliosis. The surface area occupied by, and intensity of, GFAP-positive astrocytes (green, Cy2) were increased in the corpus callosum (cc) of TGF mice and HCD-treated TGF mice compared with WT controls, as shown here in aged TGF mice (**a**) (*n* = 5–6 mice/group). Similarly, Iba-1-immunopositive microglial cells (DAB, brown) in aged TGF mice displayed an active phenotype with increased cell body and proximal processes surface area (**b**). In adult TGF mice fed a HCD, SV had no effect on either phenotype (**c**, **d**) (adult, *n* = 4 mice/group). The area of the cc used for quantitative analysis is shown by the dotted lines. Bars: 20 μm (GFAP staining); 500 μm (Iba-1 staining in top left panel); 10 μm (single Iba-1 positive microglial cells). **p < 0.01; ***p < 0.001

**Fig. 5:**
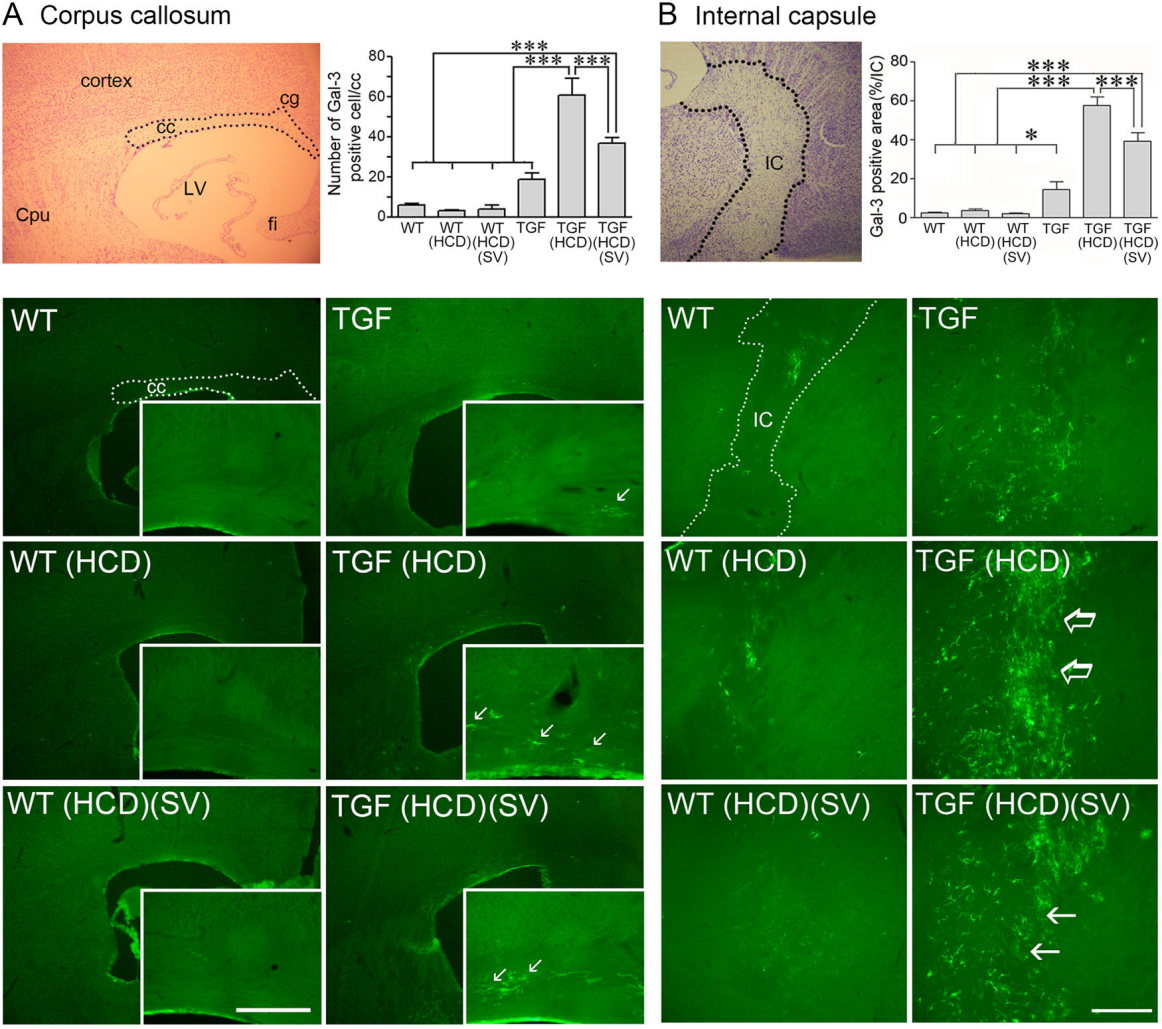
High cholesterol diet (HCD) induced white matter inflammation selectively in TGF mice, a response significantly reduced by simvastatin (SV). Cresyl-violet stained brain sections show the targeted areas (dashed line) of the corpus callosum (cc, **a**) and internal capsule (IC, **b**) used for quantitative analysis (top right panels, respectively). In TGF mice, galectin-3 (Gal-3)-immunofluorescent (green, Cy2) cell number (**a**) or surface area (**b**) was dramatically increased by HCD (arrows, right panels, respectively), an increase that was significantly reduced by SV treatment in both regions, and not seen in HCD-treated WT mice. Cpu caudate putamen, cg cingulate cortex, LV lateral ventricle, fi fibria. Bars: 200 μm (cc) and 300 μm (IC), *p < 0.05; ***p < 0.001

Astroglial and microglical cells in the cortex of TGF mice display a reactive phenotype^15,16^, which was not improved by SV^15^. Here, we found a similar reactive phenotype in the cc of adult TGF mice compared to WT mice (Fig. 4). Intensity of GFAP immunoreactive material was significantly increased, whereas both staining intensity and surface area of single Iba-1-immunopositive microglial cells were increased in the cc of TGF mice compared to WT controls (Fig. 4a, b). These phenotypes were not aggravated by HCD (Fig. 4a, b) and not reduced by SV treatment (Fig. 4c, d). In contrast to widespread Iba-1-positive microglial cells in grey and WM, we found that galectin-3 (Gal-3), a member of the galectin family of β-galactoside binding lectins found in microglia and macrophages that phagocytose myelin debris^26^, was selectively expressed in microglial cells in the WM (Fig. 5).

Gal-3 immuoreactive cells were rare in the cc and IC of adult WT mice, irrespective of being fed a HCD or not, and were slightly but not significantly more numerous in TGF mice (Fig. 5). In contrast, the number of, surface area occupied by or staining intensity (data not shown, IC only) of Gal-3 positive cells in these WM areas were significantly increased in HCD-fed TGF mice (Fig. 5). SV treatment in adult TGF mice significantly reduced this upregulating effect of HCD in both the cc (↓40% in the number of cells) and IC (↓32 and 31% decrease in surface area and staining intensity, respectively) (Fig. 5). Most WM Gal-3 positive cells displayed a microglial phenotype, as confirmed by double-immunofluorescence with Iba-1 (Supplementary Fig. 1). Immunostaining for F4/80, a marker of activated microglia and macrophages^27^, was barely detectable in the cc of WT and TGF mice, but was dramatically increased (~6 fold, *p* < 0.001) in HCD-fed TGF, but not WT, mice; a response blocked by SV (Supplementary Fig. 2).

### SV upregulated oligogenesis and MAP kinase signaling in HCD-fed TGF mice

**Fig. 6:**
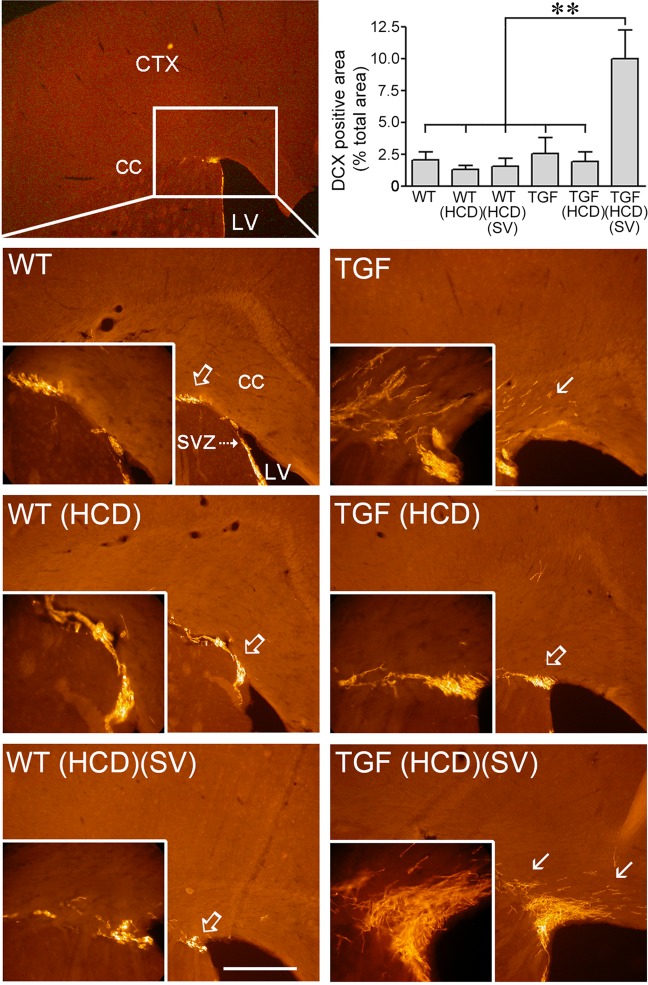
High cholesterol diet (HCD) did not alter doublecortin (DCX) positive neuroblasts in the subventricular zone (SVZ), but the area occupied by these cells was dramatically increased by simvastatin (SV) in TGF fed a HCD. A small cluster of DCX-immunostained neuroblasts (Cy3, red) was found in the rostral migratory stream (open arrow) of the SVZ in WT and TGF mice treated or not with a HCD. The surface area occupied by DCX-labelled cells in the corpus callosum (cc) was significantly larger in HCD-fed TGF mice concurrently treated with SV. The area of analysis is depicted by the white rectangle (left top panel). CTX cerebral cortex; LV lateral ventricle. Bar: 300 μm, **p < 0.01

**Fig. 7:**
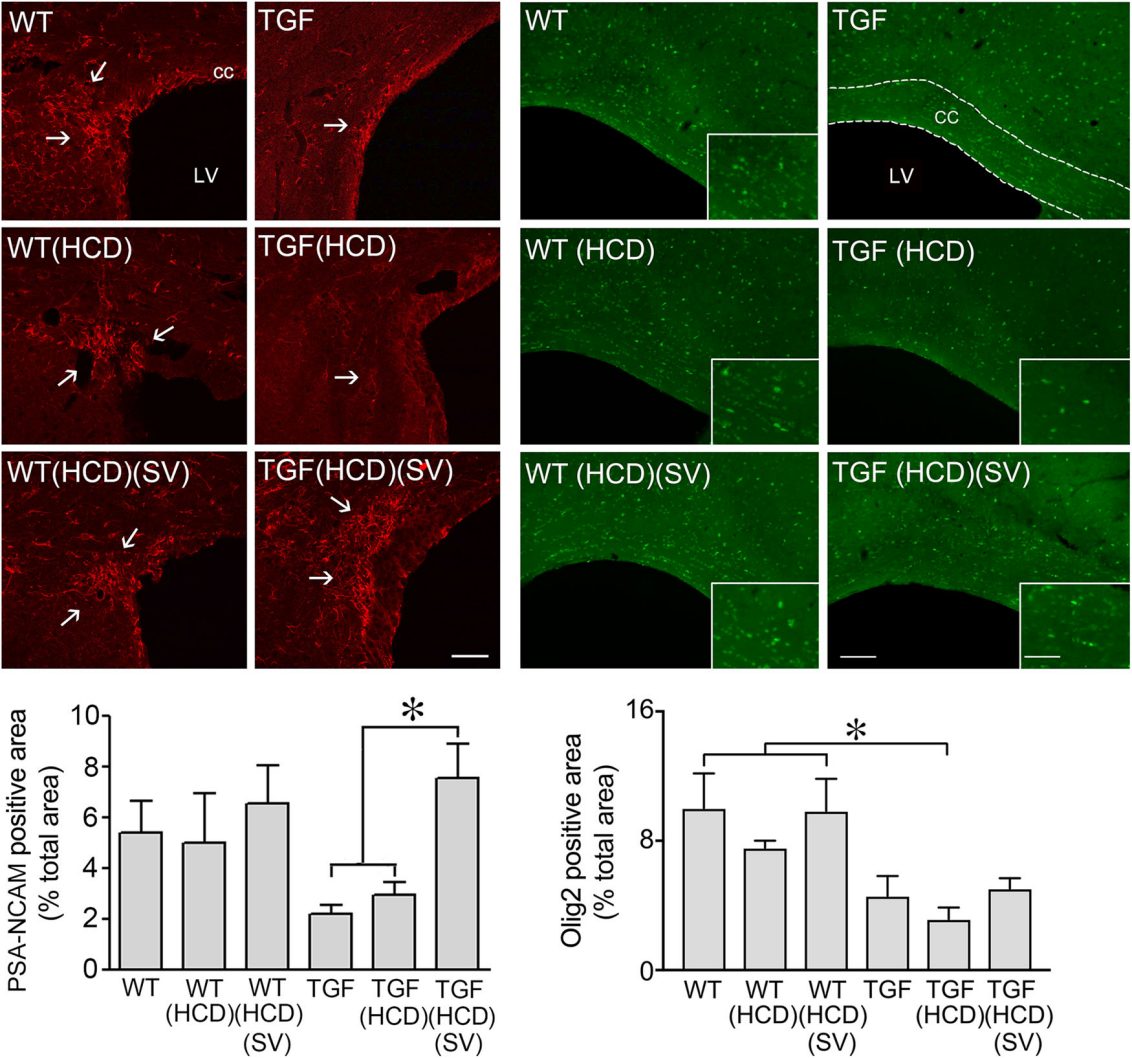
High cholesterol diet (HCD) did not alter the reduced PSA-NCAM- (left panels) and Olig2-(right panels) immunopositive material in the SVZ or corpus callosum (cc) of TGF mice: Effects of simvastatin (SV). Left: Representative confocal images showing PSA-NCAM-immunofluorescent material in the rostral migratory stream of the SVZ. Cells and thin processes immunopositive for PSA-NCAM (arrows, Alexa 594, red) occupied a comparable area of the SVZ in all groups of WT mice. In TGF and TGF mice fed a HCD, the labelled area was smaller – albeit not significantly. SV treatment in HCD-fed TGF mice increased the area to levels comparable to those seen in WT mice. Right: Olig2-immunopositive cell nuclei (green, Cy2) occupied a smaller area of the corpus callosum (cc, dotted lines) only in TGF mice fed a HCD compared to WT and HCD-fed WT mice treated with SV. HCD-fed TGF mice concurrently treated with SV did not differ from WT groups or untreated TGF mice. Bar: 500 μm, *p < 0.05

Doublecortin (DCX)-positive neuroblasts are found in the subventricular zone (SVZ) of the lateral ventricles where they normally migrate along the rostral migratory stream (RMS) to the olfactory bulb to generate interneurons. However, when demyelination occurs, DCX cells alter their properties and migratory path to become new oligodendrocytes^28^. Here, we found a cluster of DCX-immunopositive cells at the RMS-cc border of the SVZ in both WT and TGF mice, with numerous DCX-positive cells in the cc (Fig. 6). Whereas HCD did not alter this pattern, SV dramatically increased DCX-positive cells and, particularly, the density of cells in the cc in HCD-fed TGF, but not in WT mice (Fig. 6). Since progenitor cells expressing PSA-NCAM in the adult SVZ have been identified as a source of remyelinating oligodendrocytes^29^, we studied whether the RMS could be a source of newly generated oligodendrocyte progenitor cells (OPCs) for myelin repair. Our results show a reduced number, albeit not statistically significant, in PSA-NCAM-positive cells at the RMS-cc border of TGF and HCD-fed TGF mice compared to WT mice (Fig. 7). SV significantly increased PSA-NCAM immunopositive area in HCD-fed TGF mice, bringing it to levels comparable to WT mice; SV having no effect in WT mice (Fig. 7 left panel). Oligodendrocyte transcription factor 2 (Olig2) can promote differentiation of adult SVZ neuroblasts into functional oligodendrocytes^30^ and Olig2 upregulation in OPCs is a possible mechanism in myelin repair^31^. Here, although Olig2 positive cells in the cc were generally reduced in TGF compared WT mice, only HCD-fed TGF mice displayed a statistically significant decrease compared to WT groups, a similar trend being observed in HCD-fed WT mice (Fig. 7 right panel). Myelin repair having been associated with activation of the MAP kinase/ERK pathway^32^, we then investigated phospho-MAP kinase (pMAPK) immunopositive material in the cc; and it was detected only in HCD-fed TGF mice treated with SV (Supplementary Fig. 3). At the RMS-cc border, pMAPK-positive cells were intermingled but not colocalized with Gal-3 immunostained cells (Supplementary Fig. 3).

## Discussion

The most important findings from this study are: (1) HCD in TGF mice age-dependently worsened cerebrovascular dysfunction whereas it triggered cognitive deficits irrespective of age; (2) HCD’s main effect in TGF mouse brain corresponded to an inflammatory response of the WM characterized by upregulation of Gal-3-immunopositive microglial cells; (3) In HCD-fed adult TGF mice, SV treatment reduced WM inflammation, enhanced oligogenesis in the SVZ and migration of OPCs in the cc, and fully rescued both cerebrovascular and cognitive deficits without altering blood cholesterol levels. These results demonstrate that a compromised cerebral circulation, when coupled to a comorbid cardiovascular risk factor as seen in the aging population, can exacerbate cerebrovascular dysfunction, alter WM integrity and lead to cognitive failure. They further indicate that cognitive failure in this model of VCID was selectively associated with WM changes, and that both alterations could be countered by SV.

### HCD-induced cerebrovascular alterations and cognitive impairment

The age-related worsening of endothelium-dependent dilatory function and NO bioavailability in cerebral arteries of HCD-fed TGF mice together with the small harmful effect of HCD on endothelial function in WT mice indicated respective exacerbating effects of hypercholesterolemia on TGF mice with an existing compromised brain circulation and deleterious ones in WT mice, with a higher vulnerability of the aged brain vasculature. Brain endothelial cells are sensitive to high levels of circulating cholesterol and, particularly, of LDLs and oxidized LDLs that promote oxidative stress and inflammatory mediator secretion leading to impaired vasoreactivity^33,34^. Our findings concur with HC levels affecting NO bioavailability and eliciting a pro-inflammatory status in endothelial cells^35^, with consequences on NO-dependent cerebrovascular homeostasis, astrocyte differentiation^36^, angiogenesis and neurovascular coupling^37^. HCD could also affect endothelial transport of glucose, the main energy source for supporting neuronal activity. We previously found intact stimulus-induced glucose uptake in TGF mouse brain^16^, but HCD could conceivably alter this astroglial and neuronal uptake^38^.

WM disease contributes to cognitive impairment^39^, and WM alterations detected by MRI in patients with VCID have been attributed to reduced perfusion^40^, WM inflammation and/or WM degeneration^3^. We did not measure CBF in the WM, but the increased number of degenerating capillaries (string vessels) in the IC of HCD-fed TGF mice would support exacerbation of an already reduced WM perfusion in TGF mice^18^. Our findings provide arguments for endothelial cell dysfunction and cerebral hypoperfusion being aggravated by HCD^37,41^, which could lead to WM alterations^37^ and, ultimately, cognitive failure^42^. Interestingly, plasma levels of 24 s-hydroxycholesterol (24S-OHC), a brain penetrant metabolite of blood cholesterol, has been associated with cognitive deficits in hypercholesterolemic rodents^41,43^, and correlated positively with cognitive impairment in patients with VCID^44^. Overall, our findings in TGF mice agree with previous studies whereby HCD altered cognitive function only in susceptible mice with either diabetes or an amyloid pathology^45,46^. Interestingly, crossing of the same TGF mice with a mouse model of amyotrophic lateral sclerosis (SOD1^G93A^) accelerated disease progression, an effect counteracted by inhibiting TGF-β1 signaling through TGF-β type I receptors/small mothers against decapentaplegic (Smad) and non-Smad pathways^47^. Moreover, repression of TGF-β1 signaling pathway in astrocytes exerted cognitive benefits in an AD mouse model^48^. Therefore, we conclude that a susceptible brain with a cerebrovascular pathology resulting from increased brain levels of TGF-β1 as seen in TGF mice and patients with VCID^8^ may have high relevance to the pathogenesis and progression of cognitive deficits seen in VCID.

### WM alterations and cognitive deficits

In various models of WM injury^49–51^, Gal-3 was increased in activated microglial cells that phagocytose myelin debris. Recently, increased density of Gal-3 activated microglial cells in frontal WM areas of aged non-human primates was found to correlate with cognitive impairment^52^. Together, these observations support our most striking finding of selective Gal-3 positive microglial cell upregulation in the WM of cognitively impaired HCD-fed TGF mice. Gal-3 microglial cells are thought to limit WM demyelination by facilitating oligodendrocyte differentiation and, thereafter, remyelination^50,53^. It is thus tempting to suggest that the selective upregulation of WM Gal-3 microglial cells in HCD-fed TGF mice corresponded to an attempt to repair myelin damage underlying the cognitive decline. Control TGF mice displayed some WM Gal-3 microglial cell pathology that was amplified by HCD concurrently with the advent of cognitive failure, which underscored the damaging effects of HCD when combined to an existing cerebrovascular pathology in an already compromised brain. This also suggests an existing inflammatory state in the WM of TGF mice, too weak to alter cognition, but possibly increasing with age to reach a threshold sufficient to trigger the mild cognitive decline occasionally seen in aged TGF mice^20,21^. Not all Gal-3 positive cells were Iba-1 positive and F4/80-positive cells, reflecting either microglia or macrophages, were increased dramatically in the WM of HCD-treated TGF mice. Further work is needed to elucidate the potential role of infiltrating macrophages in this inflammatory response.

### WM changes and oligodendrocytes

In demyelinating conditions, DCX-expressing cells can produce oligodendrocytes in the SVZ that migrate in the cc through the RMS^28,29^. Yet, successful remyelination requires OPCs to proliferate, migrate to areas of demyelinated axons, and assume a final differentiated state. Here, albeit not significant, PSA-NCAM neuroblasts and Olig2-immunopositive cells were much less numerous in the SVZ in TGF and HCD-fed TGF mice compared to WT mice (Fig. 7), consistent with a lack of PSA-NCAM leading to less SVZ-derived cells differentiating into OPGs and migrating along the RMS^54^, two processes incidentally regulated by Gal-3^50^. These findings indicate that oligogenesis is likely impaired in TGF and HCD-fed TGF mice, although cognitive deficits were only observed in the latter group, likely due—at least partly—to their aggravated endothelial pathology. Indeed, endothelial cells play a key role in OPC generation, migration, and differentiation into oligodendrocytes, referred to as the “oligovascular niche” whereby endothelial cells secrete factors, such as vascular endothelial growth factor (VEGF) that promote oligodendrocyte migration and survival^55^. We suggest that the HCD-induced worsening of the endothelial cell pathology of TGF mice disrupted the oligovascular niche, leading to WM alterations and, consequently, cognitive deficits.

### SV treatment

SV countered the detrimental effects of HCD on cerebrovascular reactivity, including on endothelial TRPV4- and smooth muscle KATP channel-mediated dilations, and on NO bioavailability in adult TGF mice. Diet-induced hypercholesterolemia is known to cause oxidative stress and inflammation in the cerebral microvasculature^33^ even in the absence of atherosclerotic lesions^34^, and to be prevented by statin treatment in patients^33^. SV was found previously to restore NO bioavailability, NO- and KATP channel-mediated dilations in aged TGF mice^15^, dysfunctions caused by vascular inflammation^16,25^, and cerebrovascular deficits attributed to oxidative stress in an AD mouse model^22^. These clinical and pre-clinical findings point to SV being therapeutic against the cerebrovascular inflammatory pathology of TGF mice, but having the added benefit of counteracting the aggravating effects of a HCD likely related to oxidative stress.

A link between age-related cerebrovascular dysregulation, diffuse WM disease and cognitive decline has been proposed^37^. Cerebral endothelium dysfunction and WM lesions typical of VCID are more frequent in hypercholesterolemic patients than in healthy controls, and may even contribute to AD pathogenesis^56^. Here, HCD-induced worsening of the cerebrovascular pathology and WM inflammation in TGF mice occurred together with the onset of cognitive deficits, all prevented by SV. Hence, SV immunomodulatory, anti-inflammatory, and antioxidant benefits on the brain vasculature and parenchyma^22,23^ likely contributed to counter the cognitive deficits induced by HCD in TGF mice. These benefits occurred without lowering blood cholesterol, consistent with statin effects on lipid oxidation underlying their protective effects in patients with dislipidemia^57^. As in our study in aged TGF mice without cognitive deficit^15^, SV had no effect on grey matter astrogliosis and microgliosis of cognitively impaired HCD-fed TGF mice, pointing to WM alteration and recovery underlying cognitive failure and rescue.

Therefore, SV silenced the HCD-induced upregulation of Gal-3 in WM microglial cells, raising the possibility that SV can initiate myelin repair leading to cognitive recovery. In the cuprizone model of demyelination, spatial working memory was reestablished after remyelination had occurred^58^. In a mouse model of diffuse WM injury, enhanced generation of OPCs resulted in functional recovery^59^. The increased number of Olig2 cells in the cc of SV-treated mice is compatible with SV promoting oligodendrocyte differentiation^24^, SVZ-derived DCX-positive OPCs contributing to myelin repair^28,49^, and SV-mediated PSA-NCAM upregulation that is needed for efficient OPCs recruitment to demyelinated areas^29,31^.

### SV and signaling pathways in WM

Our findings of elevated pMAPK expression in the RMS of the SVZ in HCD-fed TGF mice concurrently treated with SV support a role for MAPK/ERK signaling in oligodendrocyte proliferation, migration, and maturation and, hence, remyelination^60^. Sustained activation of ERK1/2 was found sufficient to drive adult oligodendrogenesis, contribute to remyelination and enhance hippocampal-based behavior^61^. Other studies indicated that ERK signaling can reactivate quiescent mature oligodendrocytes to reinitiate myelination^32^. Our findings with SV thus support a role for MAPK/ERK pathway in myelin repair and, possibly, cognitive recovery. Cross-talk with other pathways, like the Wnt/β-catenin and Akt/mTOR pathways, however, seems likely^62,63^.

## Conclusions

Our findings highlight the relationship between HCD-induced endothelial dysfunction, WM inflammation characterized by Gal-3 upregulation in microglial cells, and cognitive failure. They further demonstrate cerebrovascular, microglial, and WM protective benefits of SV in this model of VCID, with recovery of cognitive function. In the face of an increasingly growing incidence of dementia, VCID being a main contributor, we conclude that targeting WM inflammation and, specifically, Gal-3 microglial cells, may represent a promising therapeutic avenue, as proposed in other models of demyelination accompanied with cognitive deficits^50,51^.

## Materials and methods

### Animal model and treatments

Low expresser heterozygous transgenic mice overexpressing a constitutively active form of TGF-β1 under the control of the glial fibrillary acidic protein (GFAP) promoter on a C57BL/6J background (TGF mice, line T64)^14^ were used in this study, with age-matched wild-type (WT) littermate controls. In TGF mice, TGF-β1 mRNA or protein levels are about 2–4 folds higher than in wild-type controls^15,21,64,65^, do not increase with age^21,65^, and compare well to those found in pathological brains, including AD^14,47,65^. Transgene expression was confirmed with touchdown PCR using tail-extracted DNA^15,66^. Groups of approximately equal number of males and females were used as adult (endpoint 6 months, 11–14 mice/per group) or aged (endpoint 12 months, 11−12 mice/per group) mice, and were randomized between the two treatment groups (standard or HCD). Mice were fed (3 months) either a standard diet (control) diet (4% fat, 14% protein rodent maintenance diet, Harlan Teklad global) or a HCD (supplemented with 2% cholesterol and 0.5% cholic acid, Harlan, TD. 110321). Other cohorts of adult WT and TGF mice (12−14 mice/group) received a HCD alone or concurrently with the cholesterol lowering drug simvastatin (SV, 3 months, Enzo, life science, Farmingdale, USA). SV was activated as per manufacturer’s protocol and added to the drinking water at a concentration of 0.04%, corresponding to ~40 mg/kg body weight/day^15,22^. All experiments were approved by the Animal Ethics Committee of the Montreal Neurological Institute and complied with the Canadian Council on Animal Care.

### Blood cholesterol levels

Blood samples were extracted from the heart (4−5 mice/group) in deeply anesthetized mice with isoflurane, just before they were perfused (see below). Blood was collected in Eppendorf tubes, kept at room temperature (2 h), centrifuged (15,000 rpm, 10 min) and supernatants stored (−20 °C) until use. Total cholesterol (TC), LDL and HDL cholesterol, and triglycerides were measured using a blood analyzer (Blood Research Laboratory, Royal Victoria Hospital, McGill University Health Center (MUHC), Montréal, QC, Canada).

### Morris water maze

Spatial memory was tested in the Morris water maze (MWM), as described before^15,22^. Mice first received a 3-day habituation period requiring them to swim (1.4 m diameter pool, 17 ± 1 °C opaque water) to a visible platform (60 s trials). The wall cues and platform location were then switched, the platform submerged (1 cm) and mice were submitted to 5 days of hidden-platform testing (three trials from different orientations per day, max 90 s/trial, 45 min inter-trial interval) during which mice had to find the location of the platform using distal visuo-spatial cues. On day 9, mice were given a probe trial (60 s) in which the percentage of time spent and distance traveled in the target quadrant (where the platform used to be located) were recorded, along with swim speed and the number of crossings above the previous platform location. All parameters were recorded and analyzed using 2020 Plus tracking system and Water 2020 software (HVS Image, Buckingham, UK).

### Cerebrovascular reactivity

Segments (~2 mm long) of the posterior cerebral artery (PCA, 3−5 mice/group) were cannulated, pressurized (60 mmHg) and superfused with a Krebs’ solution in a chamber for on-line videomicroscopy, as described before^15,22^. Dilations to ACh (10^−11^−10^−4^ M) and CGRP (10^−11^−10^−4^ M) were tested on vessels pre-constricted sub-maximally with phenylephrine (PE, 2 × 10^−7^M). Contractions to ET-1 (10^−11^–10^−5^ M) and the tonic production of the vasodilator NO were measured in vessels at basal tone, the latter after inhibition of NOS with L-NNA (10^−5^ M, 40 min). KATP and TRPV4 channels was assessed in pre-constricted vessels with the selective KATP (levcromakalim, LEV, 10^−11^–10^−4^ M)^22^ and TRPV4 (GSK1016790A, GSK, 10^−11^–10^−4^ M) channel openers^25^.

### Laser Doppler flowmetry

Laser Doppler flowmetry measurements (Transonic Systems Inc., Ithica, NY, USA) of increases in cerebral blood flow (CBF) evoked by whisker stimulation were performed 1 week following the MWM in a subset of anesthetized mice (4–6 mice/group, with a mixture of ketamine/xylazine (80 mg/kg; Wyeth, St-Laurent, QC, Canada/3 mg/kg; Haver, Etobicoke, ON) intramuscular) fixed in a stereotaxic frame. CBF was recorded over the left somatosensory cortex before, during and after stimulation of the right whiskers (20 s, 8−10 Hz). Four to six recordings were acquired every 30–40 s and averaged for each mouse^22^. The entire procedure lasted less than 20 min, a time window when all physiological parameters remain stable. Cortical CBF changes (peak value) were expressed as percentage increase relative to pre-stimulation baseline level.

### Total brain cholesterol levels

Measurements of brain total cholesterol levels (3−4 mice/group) were performed with the Cholesterol/Cholesteryl Ester Quantitation kit (ab65359, Abcam, Toronto, ON) according to the manufacturer. Brain tissue was homogenized in a chloroform:isopropanol:Triton x-100 solution (proportion 7:11:0.1), centrifuged (15,000 × *g*), and the resulting supernatant was dried at 50 °C to remove chloroform using speed vacuum concentration (30 min). The sample was diluted, incubated with enzyme reagents (37 °C, 60 min), and absorbance was measured (570 nm) in a microplate reader.

### Immunohistochemistry and immunofluorescence

Mice (4−6/group) deeply anesthetized with either pentobarbital (65 mg/kg) or isoflurane (mice used blood sampling) were perfused intracardially with 4% paraformaldehyde (PFA), and their brains post-fixed overnight. Then, half brains were transferred to 30% sucrose for cryoprotection, frozen (−40 °C, isopentane) and stored (−80 °C) until sectioning as free-floating sections (25 µm-thick). The other halves were processed for paraffin embedding and sectioning (5 µm-thick). For immunohistochemical staining of microglial cells, paraffin sections were incubated overnight in rabbit anti-Iba-1 (ionized calcium binding adaptor molecule-1, Wako USA, catalog # 019−19741, Richmond, 1:300), followed by biotinylated IgG (Vector lab, 1 h 30 min), ABC kit (Vector lab, 1 h 15 min), and the reaction visualized with a 0.05% DAB-Nickel solution. For single immunofluorescent staining, free-floating freezing sections were incubated overnight with either goat anti-collagen IV (Millipore, Billerica, USA, catalog # AB769, 1:300, for staining brain vessels) or anti-doublecortin (DCX- (C-18), Santa Cruz, catalog # sc-8066, 1:1000, a marker of migrating neuroblasts), rabbit anti-GFAP (Dako Canada, Burlington, Code-Nr. Z 0334, 1:2,000, a marker of astrocytes), anti-phospho MAP kinase (Erk 1/2, Cell signaling, catalog # 9101, 1:100), or anti-oligodendrocyte transcription factor 2 (Olig2, Millipore, catalog # AB9610, 1:1000, a marker of oligodendrocytes), rat anti-galectin-3 (Gal-3) (Mac-2, Cedarlane, catalog # CL8942AP, Burlington, ON, Canada, 1:1500, a marker for active WM-related microglial cells) or anti-F4/80 (Bio-Rad, catalog # MCA497, Mississauga, ON, Canada, 1:100, a marker for active microglia or macrophages), or mouse anti-polysialic acid-neural cell adhesion molecule (PSA-NCAM, Millipore, catalog # MAB5324, 1:800, protein that helps migration of oligodendrocyte precursor cells (OPC)) followed by incubation with the corresponding cyanin 2 (Cy2, green)-, Alexa 488 (green)-, cyanin 3 (Cy3, red)- or Alexa 594 (red)-conjugated secondary antibodies (Jackson laboratory, West Grove, PA, USA). For double-immunofluorescence, sections were concurrently incubated overnight with either rat anti-Gal-3 (1:1500) and rabbit anti-Iba-1 (1:300) or anti-phospho MAP kinase, followed by concurrent anti-rabbit Alexa 594 and anti-rat Alexa 488 incubation (Jackson laboratory).

### Data analysis

Vascular responses (% change diameter from basal or pre-constricted tone) were plotted as a function of agonist concentration or duration of NOS inhibition. Concentration-dependent and maximal (EAmax) responses and the agonist concentration eliciting half the EAmax (EC_50_ value or pD2 = −log EC_50_, determined with GraphPad Prism 6) were used to compare agonist efficacy and potency, respectively. String vessels were counted in the corpus callosum (cc) and internal capsule (IC) directly under the microscope by one (cc) or two independent observers (IC) blinded to the mouse identity. GFAP, Gal-3, and Olig2-positive area in the cc and IC were manually delineated on low-power digital images and measured using MetaMorph (6.1r3 software, Universal Imaging, Downington, PA) or Image J (NIH Bethesda,MD, USA). For microglial cells in the cc, 10–12 Iba-1 immunopositive single cells per mouse (4–6 mice/group) and their processes visualized in thin paraffin sections were selected randomly and their surface area measured on low-power images using MetaMorph or Image J. All data are expressed as mean ± SEM and were analyzed by two-way ANOVA (genotype and treatment as the two factors) followed by Newman-Keuls post hoc multiple comparison test or when indicated, by one-way ANOVA for TGF mice with different treatment comparisons (GraphPad Prism6). A *p* < 0.05 was considered significant.

## Electronic supplementary material

Supplemental Tables 1 and 2, Figures S1, S2 and S3.
