# Supplementary material for: High cholesterol triggers white matter alterations and cognitive deficits in a mouse model of cerebrovascular disease: benefits of simvastatin
Source: Cell Death Dis. 2019 Jan 28;10(2):89. doi: 10.1038/s41419-018-1199-0 (PMC6349936; doi:10.1038/s41419-018-1199-0)
Supplement: Supplementary file 1 — Supplemental Tables 1 and 2, Figures S1, S2 and S3. [file 41419_2018_1199_MOESM1_ESM.pdf]

High cholesterol triggers white matter alterations and cognitive deficits in a mouse model of cerebrovascular disease: benefits of simvastatin

Tong X-K, Trigiani LJ and Hamel, E

Supplemental Material

Table S1. Blood lipid levels in adult and aged WT and TGF mice fed a high cholesterol diet (HCD) with and without concurrent simvastatin (SV) treatment.

Adult

	WT (n=5)	WT (HCD) (n=4)	TGF (n=4)	TGF (HCD) (n=5)
TC	2.55±0.17	5.25±0.31***	2.15±0.75	4.54±0.32**
LDL	0.01±0.01	2.26±0.34***	0.55±0.50	2.22±0.22**
HDL	2.29±0.17	2.75±0.08	1.43±0.43	1.95±0.23
TRI	1.04±0.24	0.53±0.05	0.45±0.14	0.81±0.21

Aged

	WT (n=5)	WT (HCD) (n=4)	TGF (n=4)	TGF (HCD) (n=4)
TC	2.38±0.19	5.36±0.33***	2.44±0.11	3.81±0.16***
LDL	0.00±0.00	2.37±0.27***	0.05±0.04	1.62±0.06***
HDL	2.24±0.12	2.74±0.20	2.26±0.15	1.97±0.14
TRI	0.78±0.11	0.56±0.14	0.76±0.24	0.53±0.05

**Adult**	WT (n=4)	WT (HCD) (n=5)	TGF (n=4)	TGF (HCD) (n=5)	WT(HCD)(SV) (n=5)	TGF (HCD)(SV) (n=5)
TC	2.71±0.13	7.01±0.18***	2.73±0.26	6.12±0.28***	6.65±0.28***	6.33±0.43***
LDL	0.32±0.14	3.46±0.16***	0.45±0.08	2.96±0.40***	2.75±0.14***	2.88±0.31***
HDL	2.04±0.23	3.24±0.17**	1.88±0.27	2.90±0.09*	3.53±0.31**	3.16±0.25**
TRI	0.77±0.03	0.70±0.06	0.77±0.02	0.59±0.10	0.82±0.08	0.65±0.09

Results are expressed in (mmol/L) and are means ± SEM of the number of mice indicated within parentheses. TC: total cholesterol; LDL: low-density lipoprotein; HDL: high-density lipoprotein; Tri: triglycerides; HCD: high cholesterol diet; SV: simvastatin treatment *: $p<0.05$, **: $p<0.01$ and ***: $p<0.001$ when compared to WT using one-way ANOVA.

Table S2. Brain levels of total cholesterol (µg) per mg of cortex in adult WT and TGF mice fed a high cholesterol diet (HCD) with and without concurrent simvastatin (SV) treatment.

	WT (n=4)	WT (HCD) (n=3)	TGF (n=4)	TGF (HCD) (n=4)	WT (HCD)(SV) (n=4)	TGF (HCD)(SV) (n=4)
**Cholesterol**	9.8±2.9	15.4±4.9	16±2.5	18.2±2.8	14.8±2.5	17.1±2.9

Results are expressed in µg of total cholesterol and are means ± SEM of the number of mice indicated within parentheses.

Supplementary Figure 1

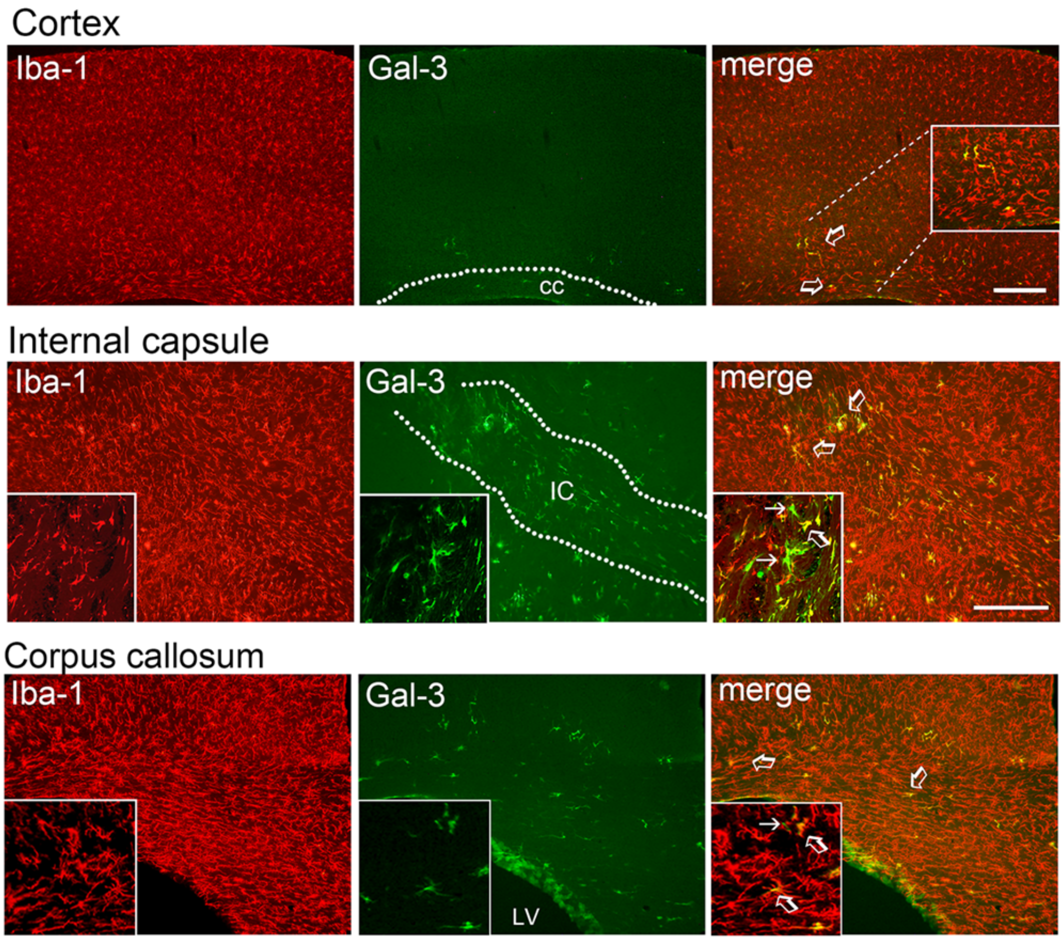

Figure S1. High cholesterol diet (HCD) in TGF mice induced increases in galectin-3 (Gal-3)-containing cells that largely co-localized with Iba-1 labeled microglial cells in both grey and white matters. In TGF mice fed a HCD, Iba-1-immunofluorescent microglial cells (Alexa 594, red, left top) distributed uniformly in the cerebral cortex where almost no Gal-3-immunofluorescence (Alexa 488, green) could be detected. The few Gal-3 positive cells found in the deep cortical layer close to the corpus callosum (cc) colocalized with Iba-1 cells (open arrows). Similarly, in the internal capsule (IC) and corpus callosum, most Gal-3-immunopositive cells co-localized with Iba-1-immunofluorescent microglial cells (yellow cells, open arrows, right panels). However, single labeled Gal-3-immunostained cells (green) were seen in both white matter areas (small arrows in insets, right panels). Bar: 300 μm; LV: lateral ventricle.

Supplementary Figure 2

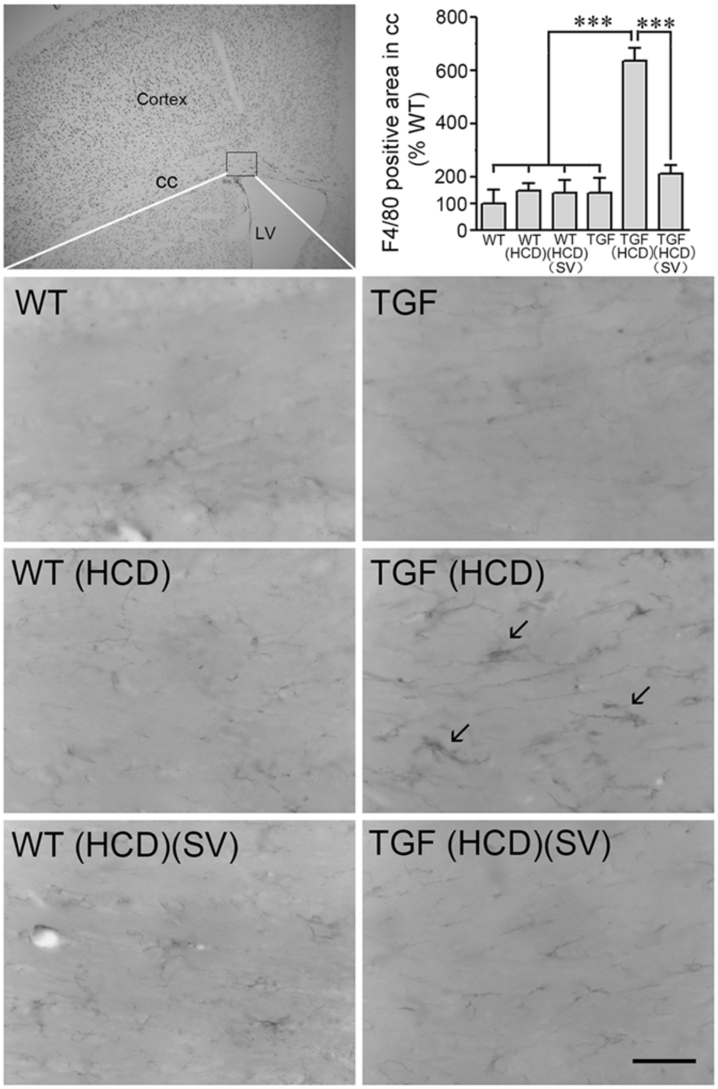

Figure S2. High cholesterol diet (HCD) selectively increased F4/80-immunolabeled material in the white matter of TGF mice: a response fully abrogated by simvastatin (SV) treatment. The surface area occupied by F4/80-immunolabeled cells in the rostral migratory stream (box in top left panel) at the junction of the corpus callosum (cc) and the subventricular zone of the lateral ventricle (LV) was drastically increased in HCD-fed TGF mice compared to all other groups. SV treatment fully countered this enhancing effect of HCD in adult TGF mice. Bar: 50 μm, ***: $p < 0.001$.

Supplementary Figure 3

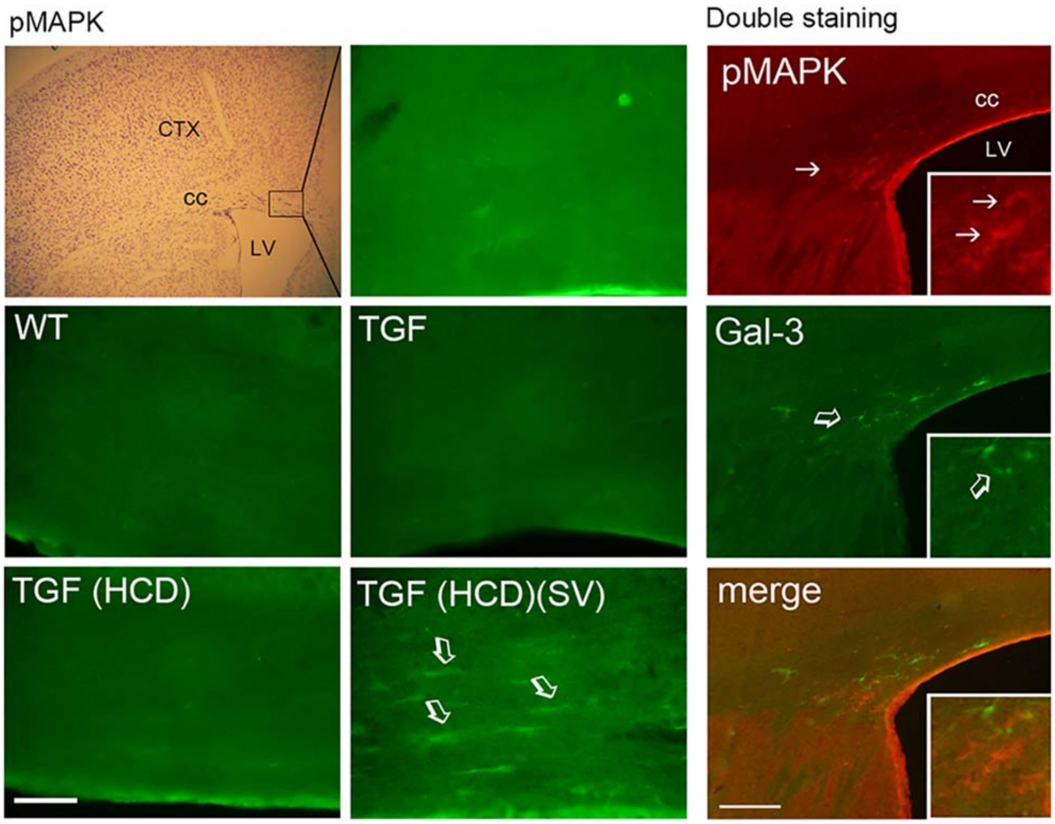

Figure S3. Simvastatin (SV) upregulated phospho-MAP kinase (pMAPK) in the white matter of HCD-fed TGF mice. pMAPK-immunostained cells (Cy2, green) could be detected only in the corpus callosum (cc) of HCD-fed TGF mice treated with SV (open arrows, bottom of middle panels). Double-immunostaining (right panels) of pMAPK (red, small arrows) and galectin-3 (Gal-3, green, open arrows) showed that the upregulation of pMAPK induced by SV occurred in cells that were immunonegative for Gal-3 (merge, bottom right panel and inset). Bars: 50 μm (left panel), 200 μm (right panel). CTX: cerebral cortex; LV: lateral ventricle.
